# The association between body image and depressive symptoms in pregnant and postpartum women: a meta-analysis

**DOI:** 10.3389/fpubh.2025.1655639

**Published:** 2025-10-08

**Authors:** Jingbo He, Xin Chen, Biru Luo

**Affiliations:** ^1^Department of Nursing, West China Second University Hospital, Sichuan University/ West China School of Nursing, Sichuan University, Chengdu, Sichuan, China; ^2^Key Laboratory of Birth Defects and Related Diseases of Women and Children (Sichuan University), Ministry of Education, Chengdu, Sichuan, China; ^3^Department of Obstetrics Nursing, West China Second University Hospital, Sichuan University/ West China School of Nursing, Sichuan University, Chengdu, Sichuan, China

**Keywords:** body image, depression, pregnancy, postpartum period, body dissatisfaction

## Abstract

**Background:**

Body image is an individual’s internal representation of physical appearance. Perinatal depression, a psychological condition with severe implications, is influenced by body dissatisfaction. However, no studies have systematically quantified their association.

**Objective:**

To evaluate the correlation between body image and depressive symptoms in the perinatal period through meta-analysis.

**Methods:**

A systematic search was conducted in PubMed, Embase, The Cochrane Library, Medline, CNKI, Wanfang, and VIP databases, and studies assessing body image and perinatal depression were included. Two researchers independently screened, extracted, and assessed study quality. Meta-analysis used Review Manager 5.4, with the correlation coefficient (r) as the effect size, and studies assessing body image and perinatal depression were included.

**Results:**

Twenty-eight studies involving 7,241 women were included. For pregnancy, the summary r for the reverse and forward scoring groups was 0.34 (95% CI: 0.24, 0.44; *p* < 0.01) and −0.34 (95% CI: −0.37, −0.30; *p* < 0.01), respectively. The four dimensions of body image (Feeling Fat, Attractiveness, Salience of Weight and Shape, and Strength and Fitness) were 0.34 (95% CI: 0.28, 0.40), −0.36 (95% CI: −0.42, −0.31), 0.31 (95% CI: 0.25, 0.36), and −0.32 (95% CI: −0.37, −0.26), respectively (all *p* < 0.01). For postpartum, the summary *r* = 0.35 (95% CI: 0.26, 0.43; *p* < 0.01) (reverse scoring group), and for the four dimensions: 0.30 (95% CI: 0.23, 0.38), −0.41 (95% CI: −0.46, −0.36), 0.27 (95% CI: 0.20, 0.35), and −0.34 (95% CI: −0.39, −0.28), respectively (all *p* < 0.01). The results for all subgroups were robust, with no significant publication bias.

**Conclusion:**

Body dissatisfaction is consistently and moderately associated with perinatal depression. Early identification and interventions may help prevent depression and improve maternal–infant health outcomes.

**Systematic review registration:**

PROSPERO, identifier CRD42025639158, https://www.crd.york.ac.uk/PROSPERO/view/CRD42025639158.

## 1 Introduction

The perinatal period is a unique phase in women’s lives, characterized by multiple transitions in physical, psychological, and social roles. During this period, they not only undergo significant changes but also face psychological challenges associated with identity transformation (1, 2), making it a vulnerable stage for the development of mental health problems (3). Perinatal depression, defined as depression occurring during pregnancy or postpartum, is a particularly pressing mental health concern. Data from the United States Centers for Disease Control and Prevention indicated that pregnancy-related deaths caused by mental health disorders accounted for 22.7% of deaths from 2017 to 2019 in the U. S., making it a major contributor to perinatal mortality (4). Beyond impairing maternal mental health, perinatal depression exerts far-reaching implications on offspring and families. Specifically, pregnancy-related depression may increase the risk of adverse outcomes, such as fetal growth restriction, preterm birth, and low birth weight; elevate the incidence of postpartum depression and spousal depression; and reduce breastfeeding rates (5). Postpartum depression can disrupt the mother-infant bonding (6), which may, in turn, impair the normal development of children’s emotions, cognition, and behaviors (7) and lead to maternal role maladaptation and decreased quality of life (6).

Beyond the impacts, previous studies have identified multiple risk factors for perinatal depression, including a history of depression, inadequate social and economic support, chronic stressful life events, unintended pregnancies (8), and traumatic childbirth experiences (9). In recent years, body image has emerged as an increasingly recognized factor influencing perinatal mental health. Growing evidence links body image to several perinatal outcomes, such as postpartum anxiety, depression, eating disorders, mother-infant bonding, and other problems (10–13). Most of these studies were conducted in Western contexts, with relatively fewer studies from Asian populations.

Body image is defined as an individual’s internal representation of their physical appearance, encompassing three dimensions: cognition, affective, and behavioral intention (14, 15). While societal ideals of female beauty have evolved over time, women consistently face pressure to conform to the mainstream aesthetics of their respective societies. Chronic social comparison of one’s appearance to these societal ideals may lead to cognitive biases, which in turn may result in body image disturbance or body dissatisfaction—negative perceptions and evaluations of one’s physical appearance (14, 15). Existing research has established a moderate-to-strong association between body dissatisfaction and adverse mental health outcomes, including depression, anxiety, and distress (16). Although pregnancy is often perceived as a “protected” phase, growing evidence emphasizes that sociocultural pressures persist, driving women to pursue unrealistic bodily and aesthetic ideals (2, 17).

During pregnancy, rapid physiological changes in women’s bodies may further diverge from societal ideals of body shape, prompting the reassessment of body image (18). Evidence from the United States suggests that over 50% of perinatal women experience body dissatisfaction (19, 20). Unlike the non-pregnant states, the changes during pregnancy are normal physiological adaptations to support fetal development. U.S.-based research investigating behaviors associated with positive body image among postpartum mothers has shown that some women prioritize maternal bodily function over aesthetic concerns, facilitating adaptive re-evaluation of their body image and psychological adjustment (10, 21). In contrast, others struggle to accept these changes, experiencing heightened awareness of altered physical appearance and negative emotions toward their bodies—even perceiving these changes as threats to their self-identity (22). Negative body image during pregnancy has been linked to a range of adverse maternal and child health outcomes, most prominently pregnancy and postpartum depression (17, 23–25). Beyond this, negative body image is also significantly associated with impaired emotion regulation (26). For instance, a French study found that women with body dissatisfaction were four times more likely to develop perinatal depression (27). Similarly, a large longitudinal study from Hong Kong, China, indicated that body dissatisfaction may either precede depression and anxiety (25) or serve as a somatic manifestation of emotional symptoms. Given these associations, assessing body image could serve as an effective tool for early identification of perinatal depression.

Although many studies and narrative reviews have explored and summarized the role of body image in perinatal depression, they did not provide a quantitatively synthesized effect size or examine stage-specific differences. Most of the existing evidence, particularly from countries such as Australia and the United States, is based on individual observational studies, making it difficult to draw consistent conclusions. Therefore, a meta-analytic approach is needed to integrate the fragmented evidence and provide a precise quantitative estimate of this association. Accordingly, the aim of this review is to (1) systematically assess and quantify the association between body image and perinatal depression; (2) explore potential differences in the strength of this association between the pregnancy and postpartum periods through subgroup analysis; and (3) provide evidence to increase healthcare professionals’ attention to body shape and weight-related pressures experienced by perinatal women, thereby supporting the integration of body image assessment into routine perinatal care.

## 2 Materials and methods

This meta-analysis is registered in the International Prospective Register of Systematic Reviews (PROSPERO) trial registry (CRD42025639158) and was conducted in accordance with the Preferred Reporting Items for Systematic Reviews and Meta-Analyses (PRISMA) guidelines.

### 2.1 Search strategy

The following databases were searched to identify relevant research: PubMed, Embase, The Cochrane Library, Medline, China National Knowledge Infrastructure (CNKI), Wanfang Database, and VIP Chinese Journal Database (VIP). Additionally, the reference lists of included studies were checked for a complete literature search. Searches covered the period from the inception of each database to December 2024. The search strategies are presented in Supplementary Data 1.

A combination of subject terms and free-text terms was used for the search:

Subject Terms: Pregnancy, Pregnancy Trimester; Body Image, Body Dissatisfaction; Depression, Postpartum, Puerperal Disorders, Depressive Disorder/Postnatal Depression.

Free-text Terms: pregnant*, gravida*, matern*, gestation, prenatal, antenatal; body satisfaction, body appreciation, body concerns, body image disturbance, body schema*, body representation*; postpartum depression, postnatal depression, depression, postnatal, perinatal depression, puerperium depression, new mother depression, maternal depression, postpartum mood disorders, depression after childbirth, post-birth depression, baby blues.

### 2.2 Selection criteria

The inclusion and exclusion criteria for this study were as follows:

Inclusion Criteria:

1. Study population: women during pregnancy or the postpartum period;
2. Outcome measurements: assessment of both body image and depressive symptoms during pregnancy or the postpartum period;
3. Statistical analysis: reporting of correlation analysis between the body image and depressive symptoms;
4. Language: studies published in Chinese or English.

Exclusion Criteria:

1. Outcome measurement: Use of self-designed questionnaires without validation.
2. Accessibility: unavailability of the full text.
3. Data availability: failure to report or extract specific correlation coefficients.

### 2.3 Literature screening

Retrieved records were first de-duplicated using EndNote X9 software. Two independent authors then screened the titles and abstracts to exclude ineligible studies, including animal studies, reviews, systematic reviews, meta-analyses, qualitative studies, and case reports based on titles and abstracts. After reading the full text, studies with irrelevant content or those lacking correlation coefficients were further excluded. Any disagreements were resolved through discussion with the research team.

### 2.4 Quality assessment

Two independent authors used the Agency for Healthcare Research and Quality (AHRQ) checklist to assess the quality of the included studies. Referring to a previous meta-analysis (28), each item of the AHRQ checklist was scored as 1 point (for “yes” responses) or 0 points (for “no” or “unclear” responses). A total score of 0–3 was categorized as low quality, 4–7 as medium quality, and 8–11 as high quality. The quality assessment process was the same as the literature screening.

### 2.5 Data extraction

Two independent authors extracted data from each included study: the first author, publication year, country, study design, sample size, maternal age, timing of outcome measurement, measurement tools and scores, and correlation coefficients between body image and depression symptoms.

### 2.6 Outcome measures

#### 2.6.1 Body image levels

The body image was assessed using two categories of measurement tools: pregnancy-specific tools and universal tools.

Pregnancy-specific tools: The Body Understanding Measure for Pregnancy Scale (BUMPs) (29) and the Body Image in Pregnancy Scale (BIPS) (30).

Universal tools: The Body Attitudes Questionnaire (BAQ) (31), Body Shape Questionnaire (BSQ) (32), Eating Disorder Inventory (EDI) (33), Body Cathexis Scale (BCS) (34), Body Image Concern Inventory (BICI) (35), Body Areas Satisfaction Scale (BASS) (36), and Body Self Questionnaire (BSQ-self) (37). Among these tools, the Body Part Satisfaction Scale (BPSS) (38), Body Image Scale (BIS) (39), and BASS (36) are scored in the forward direction; higher scores indicate greater body satisfaction, while the other tools are scored in the reverse direction; higher scores indicate greater body dissatisfaction.

#### 2.6.2 Depression levels

Depressive symptoms were assessed using the following tools: Edinburgh Postnatal Depression Scale (EPDS) (40), Beck Depression Inventory (BDI) (41), Hospital Anxiety and Depression Scale (HADS) (42), Center for Epidemiologic Studies Depression Scale (CES-D) (22), and the Depression subscale of the Depression, Anxiety, and Stress Scale (DASS) (43).

### 2.7 Data analysis

Statistical analyses were performed using Review Manager 5.4, with a significance level set at *p* < 0.05.

Heterogeneity was assessed using Cochran’s *Q* test and the *I*^2^ statistics (44–46). The pooled effect size was estimated based on heterogeneity results: a fixed-effects model was used if *p* > 0.1 and *I*^2^ < 50% (low-to-moderate heterogeneity), whereas a random-effects model was used if *p* < 0.1 and *I*^2^ ≥ 50% (high heterogeneity).

Pearson’s correlation coefficient (r) was used as the effect size to quantify the association between body image and depressive symptoms. For meta-analysis, (1) Spearman’s correlation coefficients from individual studies were first converted to Pearson’s *r*. (2) All Pearson’s *r* values were transformed using Fisher’s *z*-transformation. (3) Inverse transformation of Fisher’s *z*-scores was performed to estimate the summary correlation coefficient (summary *r*) (47). The detailed formulas are provided in Supplementary Data 2. When multiple subgroup results were reported in one study, each result was treated as an independent study.

Subgroup analyses were conducted according to the timing of outcome measurement (pregnancy/postpartum) and the scoring direction of body image tools (forward/reverse scoring). Sensitivity analysis was performed using the leave-one-out method to evaluate the robustness of the pooled result. Publication bias was assessed using funnel plots and the trim-and-fill method in R 4.4.2. The detailed R codes are provided in Supplementary Data 3.

## 3 Results

### 3.1 Literature search results

**Figure 1:**
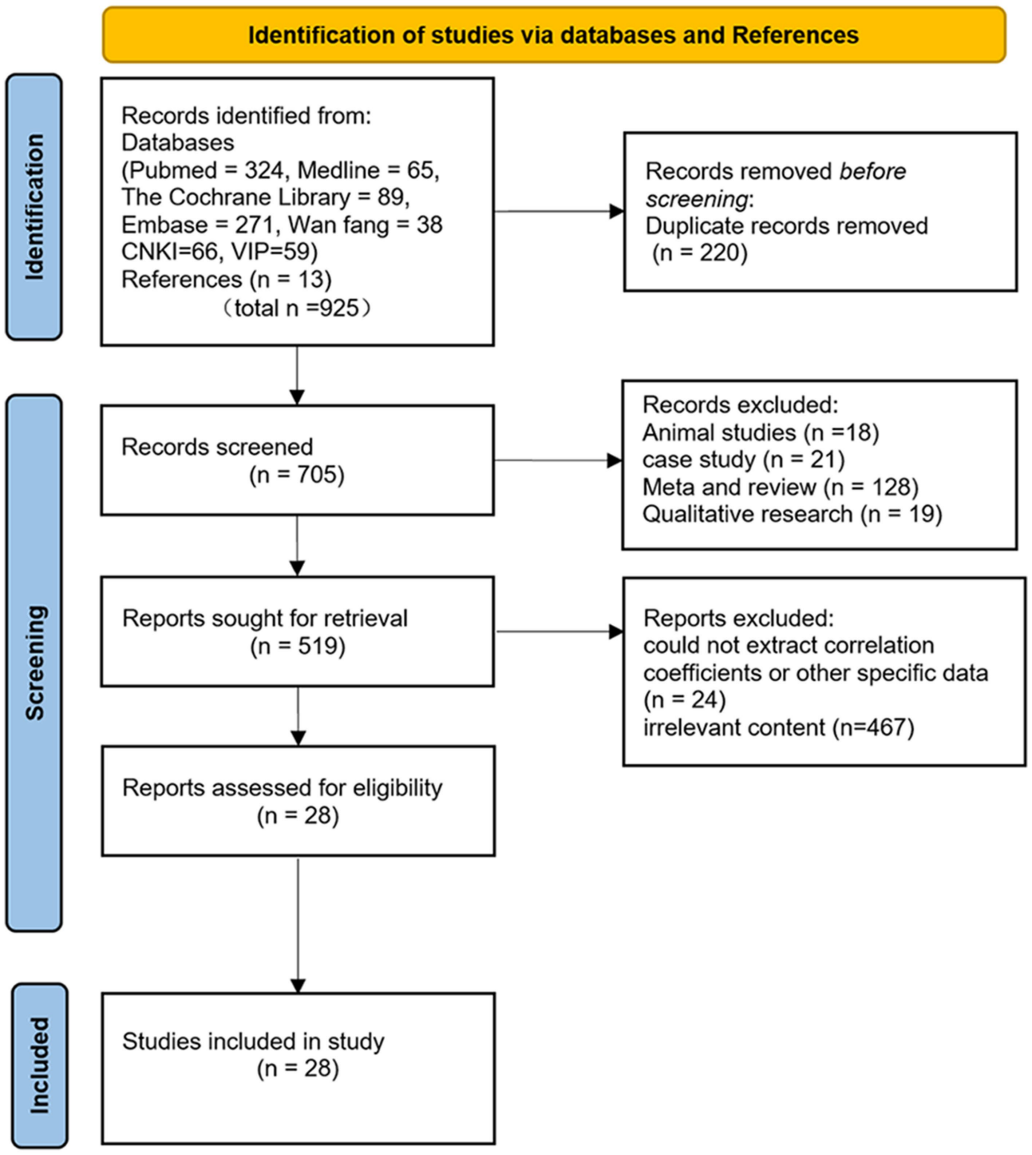
PRISMA flowchart of study selection.

The results of the literature search and screening process are illustrated in Figure 1 (PRISMA flowchart). A total of 925 records were retrieved from the databases. After removing 220 duplicates, 705 records were screened based on titles and abstracts. During this screening stage, 186 records were excluded (including animal studies, narrative reviews, systematic reviews, meta-analyses, case reports, and qualitative studies), leaving 519 records for full-text assessment. After full-text review, 467 records were excluded due to irrelevant content, and 24 records could not extract correlation coefficients or other specific data. Ultimately, 28 articles that met the inclusion and exclusion criteria were included in the meta-analysis (17, 39, 48–73).

### 3.2 Characteristics of included studies

The 28 included studies comprised a total sample of 7,241 women. Seventeen were cross-sectional studies (17, 39, 48–51, 53–55, 60–62, 65, 67, 70–72), and 11 were longitudinal (52, 56–59, 63, 64, 66, 68, 69, 73). Geographically, the majority of studies were conducted in Australia (*n* = 11) (17, 48, 55, 57–59, 61, 63, 64, 66, 69), followed by the United States (*n* = 7) (51–54, 56, 60, 68), and 3 from China (49, 72, 73). The studies covered three time periods: pre-pregnancy, pregnancy, and postpartum. Seventeen studies reported the correlation coefficients for body image and depression during pregnancy (17, 39, 48, 51, 54–57, 59, 62, 65–68, 70–72), eleven reported the correlation coefficients for postpartum body image and postpartum depression (49, 50, 52, 53, 57–61, 64, 70), one study reported the correlation coefficients for pre-pregnancy body image and postpartum depression (63), two reported the correlation coefficients for pre-pregnancy body image and pregnancy depression (66, 69), and two reported the correlation coefficients for pregnancy body image and postpartum depression (56, 73). For depression assessment, the Edinburgh Postnatal Depression Scale (EPDS) was the most widely used tool (*n* = 14) (17, 48–50, 53, 54, 58, 59, 61, 62, 64, 70, 72, 73). For body image measurement, the Body Attitudes Questionnaire (BAQ) was the most frequently used (*n* = 9) (55, 57–59, 63, 64, 66, 69, 71) (Table 1).

**Table 1:** Characteristics of the included studies (*n* = 28).

Author publication year	Country	Study design	Sample size	Age	Timing of outcome measurement	Body image Measurement tool	Scores	Depression measurement tools	Scores	Correlation coefficients	Spearman’s r converted to Pearson’s r	Quality of included studies
Adele Samra (2024) (48)	Australia	Cross-sectional	231	31.91 ± 4.39	Pregnancy	BIPS	93.75 ± 19.54	EPDS	8.76 ± 4.44	Spearman’s *r*: *r* = 0.50, *p* < 0.01	*r* = 0.52, *p* < 0.01	6 middle
Yang, Yiyun (2024) (73)	China	Longitudinal	362	≤30, 194 cases; >30, cases	1. Late pregnancy (3–4 Days before delivery) 2. 14 Days postpartum	BIPS	89.24 ± 15.56	EPDS	7.50 (4.00, 11.00)	Pearson’s *r*: *r* = 0.402, *p* < 0.001		6 middle
Fan-Hao Chou (2004) (51)	USA	Cross-sectional	113	27.06 ± 4.42	6–10 weeks of pregnancy	BCS	/	CES-D	/	Spearman’s *r*: *r* = 0.21, *p* < 0.05	*r* = 0.22, *p* < 0.05	5 middle
Alissa Haedt (2007) (54)	USA	Cross-sectional	188	28.75 ± 55.40	Pregnancy	BSQ	22.28 ± 8.76	EPDS	7.62 ± 4.08	Pearson’s *r*: *r* = 0.39, *p* < 0.001		5 middle
Hanna Przybyła-Basista (2020) (62)	Poland	Cross-sectional	150	27.83 ± 4.60	Pregnancy	BSQ-self	Median: 16	EPDS	Median: 8	Pearson’s *r*: *r* = 0.629, *p*<0.01		6 middle
Ekaterina Kamysheva (2008) (55)	Australia	Cross-sectional	215	31.73 ± 4.54	15–25 weeks of pregnancy	BAQ	BAQ FF: 30.26 ± 9.40 BAQ Attr: 16.31 ± 3.14 BAQ Sal: 11.33 ± 3.15 BAQ SFit: 17.97 ± 4.20	BDI	1.68 ± 0.84	Pearson’s r: BAQ FF: 0.29, *p* < 0.05; BAQ Attr: −0.32, *p* < 0.05; BAQ Sal: 0.29, *p* < 0.05; BAQ SFit: −0.34, *p* < 0.05		5 middle
Dianne Duncombe (2008) (66)	Australia	Longitudinal	158	31.7 ± 3.7	1. Pre-pregnancy (Retrospective) 2. 16–23 weeks of pregnancy 3. 24–30 weeks of pregnancy 4. 6–10 weeks of pregnancy	BAQ	T1: BAQ FF: 31.18 ± 10.72 BAQ Attr: 17.67 ± 2.53 BAQ Sal: 11.99 ± 4.12 BAQ SFit: 20.17 ± 4.24 T2: BAQ FF: 32.81 ± 11.06 BAQ Attr: 17.27 ± 3.25 BAQ Sal: 11.49 ± 3.80 BAQ SFit: 18.51 ± 4.03 T4: BAQ FF: 30.41 ± 10.55 BAQ Attr: 17.33 ± 3.23 BAQ Sal: 10.70 ± 3.49 BAQ SFit: 19.26 ± 3.98	BDI	T2: 3.42 ± 3.04 T3: 3.71 ± 2.90	Pearson’s *r*: Pre-pregnancy Body Image and Depression in First Pregnancy: BAQ FF: 0.31, *p* < 0.001; BAQ Attr: −0.20, *p* < 0.01; BAQ Sal: 0.24, *p* < 0.01 Pre-pregnancy Body Image and Depression in Second Pregnancy: BAQ FF: 0.28, *p* < 0.001 Body Image and Depression in First Pregnancy: BAQ FF: 0.37, *p* < 0.001; BAQ Attr: −0.39, *p* < 0.001; BAQ Sal: 0.31, *p* < 0.001; BAQ SFit: −0.34, *p* < 0.001 Body Image in First Pregnancy and Depression in Third Pregnancy: BAQ FF: 0.32, *p*<0.001; BAQ Attr: −0.29, *p* < 0.001; BAQ Sal: 0.31, *p* < 0.001; BAQ SFit: −0.30, *p* < 0.001 Body Image in Third Pregnancy and Depression: BAQ FF: 0.45, *p* < 0.001; BAQ Attr: −0.46, *p* < 0.001; BAQ Sal: 0.38, *p* < 0.001; BAQ SFit: −0.26, *p* < 0.01		3 low
Helen Skouteris (2005) (69)	Australia	Longitudinal	128	31.63 ± 3.44	1. First 3 months of pregnancy (Retrospective) 2. 16–23 weeks of pregnancy 3. 24–31 weeks of pregnancy 4. 32–39 weeks of pregnancy	BAQ	T2: BAQ FF: 32.82 ± 10.10 BAQ Attr: 17.80 ± 2.72 BAQ Sal: 11.77 ± 3.76 BAQ SFit: 21.11 ± 4.31 T4: BAQ FF: 30.95 ± 9.82 BAQ Attr: 17.15 ± 3.40 BAQ Sal: 11.26 ± 3.37 BAQ SFit: 19.52 ± 3.95	BDI	3.26 ± 2.58	Pearson’s *r*: Pre-pregnancy Body Image and Depression in First Pregnancy: BAQ FF:0.47, *p* < 0.001; BAQ Attr: −0.20, *p* < 0.05; BAQ Sal: 0.35, *p* <0.05		6 middle
Juliana Meireles (2017) (71)	Brazil	Cross-sectional	386	29.32 ± 6.04	Pregnancy	BAQ	121.39 ± 17.30	BDI	10.86 ± 8.84	Pearson’s *r*: *r* = 0.387, *p* < 0.05		4 middle
Rachel Dryer (2020) (17)	Australia	Cross-sectional	408	28.24 ± 5.04	Pregnancy	BPSS	44.66 ± 10.69	EPDS	10.16 ± 5.64	Pearson’s *r*: *r* = −0.37, *p* < 0.05		3 low
Kranti S. Kadam (2023) (65)	India	Cross-sectional	146	26.40 ± 4.07	Pregnancy	BICI	23.15 ± 10.43	BDI	8.67 ± 14.04	Spearman’s *r*: *r* = 0.1854, *p* < 0.05	*r* = 0.19, *p* < 0.05	5 middle
Esra Cevik (2019) (39)	Turkey	Cross-sectional	362	26.0 ± 5.1	Pregnancy ≥ 28 week	BIS	148.2 ± 22.4	BDI	10.4 ± 6.5	Spearman’s r: *r* = −0.291, *p* = 0.001	*r* = −0.30, *p* = 0.001	5 middle
Lydia Beatrice Munns (2024) (67)	UK	Cross-sectional	253	31.9 ± 5.0	Pregnancy	BUMPs	59.7 ± 13.6	HADS	5.7 ± 3.1	*β* = 0.093, *p* < 0.01	*r* = 0.093, *p* < 0.01	5 middle
Zhang Xuan (2022) (72)	China	Cross-sectional	946	30.56 ± 4.03	Pregnancy	BUMPs	/	EPDS	/	Pearson’s *r*: *r* = 0.246, *p* < 0.001		5 middle
Erica L. Rauff (2011) (68)	USA	Longitudinal study	151	30.2 ± 4.0	1. 14 weeks of pregnancy 2. 21 weeks of pregnancy 3. 32 weeks of pregnancy	BASS	T1: 27.9 ± 4.7 T2: 28.2 ± 4.8 T3: 28.0 ± 4.7	CES-D	T1: 11.8 ± 8.9 T2: 10.2 ± 8.3 T3: 12.0 ± 9.0	Body Image in First Trimester and Depression in Second Trimester: *β* = −0.22, *p* < 0.05 Body Image in Second Trimester and Depression: *β* = −0.31, *p*<0.001	*r* = −0.22, *p* < 0.05 *r* = −0.31, *p*<0.001	5 middle
Danielle Symons Downs (2008) (56)	USA	Longitudinal	230	30.05 ± 4.13	1. First Trimester 2. Second Trimester 3. Third Trimester 4. 6 weeks postpartum	BASS	T1:27.5 ± 4.8 T2:27.6 ± 5.2 T3:27.5 ± 5.6 T4:25.5 ± 5.2	CES-D	T1:10.9 ± 7.8 T2:9.7 ± 7.8 T3:10.4 ± 7.4 T4:9.9 ± 8.1	Pearson’s r: First Trimester: −0.36, *p* < 0.01; Second Trimester: −0.37, *p* < 0.01; Third Trimester: −0.41, *p* < 0.01 Body Image in First Pregnancy and Depression in Second/Third Pregnancy: −0.33, −0.24, all *p* < 0.01 Body Image in Second Pregnancy and Depression: −0.41, *p* < 0.01 Body Image in First/Second/Third Pregnancy and Postpartum Depression: −0.36, –0.48, –0.43, all *p* < 0.01		6 middle
Mei-Ling Chen (2023) (49)	Taiwan, China	Cross-sectional	330	20–25: 15.7% 26–30: 27.6% 31–35: 38.8% >35: 17.9% (Proportion of Each Age Group)	4–6 weeks postpartum	BASS	27.27 ± 6.71	EPDS	EPDS≦9: 60% EPDS<9: 40% (Stratified Proportion)	Spearman’s *r*: *r* = −0.21, *p*<0.01	*r* = −0.22, *p* < 0.01	5 middle
Francisco Javier Riesco-González (2022) (50)	Spain	Cross-sectional	449	31.99 ± 5.829	Within 6 months postpartum	BSQ	20.60 ± 8.9	EPDS	8.65 ± 5.67	Spearman’s *r*: *r* = 0.42, *p* < 0.001	*r* = 0.44, *p* < 0.001	4 middle
Megan F. Lee (2019) (61)	Australia	Cross-sectional	419	32.06 ± 5.30	6–48 months postpartum	BSQ-self	50.69 ± 18.60	EPDS	15.96 ± 10.99	Pearson’s r: r = 0.52, *p*<0.001		6 middle
Grazia Terrone (2023) (70)	Italy	Cross-sectional	170	41.17 ± 5.23 40.51 ± 5.59	1. pregnancy 2. after delivery	BSQ	T1: 69 ± 33.42 T2: 96.18 ± 47.3	pregnancy: BDI; After Delivery: EPDS	T1: 9.194 ± 7.108 T2: 17.52 ± 6.56	Pearson’s *r*: 1. Body Image and Depression in Pregnancy: *r* = 0.328, *p*<0.05 2. Body Image and Depression after Delivery: *r* = 0.354, *p*<0.05		4 middle
Lorraine Walker (2002) (52)	USA	Longitudinal	283	Anglo/White: 22.8 ± 4.4 African American: 22.2 ± 3.8 Hispanic: 21.9 ± 3.4	1. after delivery 2. 6 weeks postpartum	BCS	T1: 68.6 ± 19.0 T2: 68.9 ± 18.2	CES-D	T1: 22.4 ± 10.2 T2: 20.2 ± 11.2	Pearson’s r: 1. Body Image and Depression after Delivery: 0.34, *p* < 0.001 2. Body Image after Delivery and Depression at 6 Weeks Postpartum: *r* = 0.19, *p* < 0.01 3. Body Image at 6 Weeks Postpartum and Depression after Delivery: *r* = 0.28, *p* < 0.001 4. Body Image and depression at 6 Weeks Postpartum: *r* = 0.3, *p* < 0.001		6 middle
Robyn Birkeland (2005) (53)	USA	Cross-sectional	149	17 ± 1.03	3–12 months postpartum	EDI	14.22 ± 6.1	EPDS	9.65 ± 5.40	Pearson’s *r*: *r* = 0.29, *p*<0.01		4 middle
Abigail Clark (2009) (57)	Australia	Longitudinal	116	31.78 ± 3.71	1. First Trimester (Retrospective) 2. 17–21 weeks of pregnancy 3. 32–35 weeks of pregnancy 4. 6 weeks postpartum 5. 6 months postpartum 6. 12 months postpartum	BAQ	T1: BAQ FF: 32.89 ± 10.19 BAQ Attr: 18.01 ± 2.89 BAQ Sal: 12.29 ± 4.14 BAQ SFit: 20.56 ± 4.60 T2: BAQ FF: 32.71 ± 10.77 BAQ Attr: 17.55 ± 3.31 BAQ Sal: 11.68 ± 3.87 BAQ SFit: 18.66 ± 4.17 T3: BAQ FF: 29.63 ± 9.70 BAQ Attr: 10.34 ± 3.08 BAQ Sal: 12.61 ± 1.92 BAQ SFit: 19.26 ± 4.29 T4: BAQ FF: 36.34 ± 11.27 BAQ Attr: 17.90 ± 3.50 BAQ Sal: 11.63 ± 4.15 BAQ SFit: 19.93 ± 4.23 T5: BAQ FF: 37.45 ± 11.97 BAQ Attr: 17.39 ± 3.68 BAQ Sal: 12.25 ± 4.55 BAQ SFit: 20.39 ± 4.72	BDI	T2.3.15 ± 2.58 T3.3.96 ± 3.63 T4.3.29 ± 2.88 T5.3.23 ± 3.21 T6.2.86 ± 2.75	Pearson’s *r*: T1: BAQ FF: 0.34, *p* < 0.01; BAQ Attr: −0.36, *p* < 0.01; BAQ SFit: −0.36, *p* < 0.01 T2: BAQ FF: 0.31, *p* < 0.01; BAQ Attr: −0.44, *p* < 0.01; BAQ Sal: 0.23, *p* < 0.05; BAQ SFit: −0.30, *p* < 0.05 T3: BAQ FF: 0.43, *p* < 0.01; BAQ Attr: −0.44, *p* < 0.01; BAQ Sal: 0.42, *p* < 0.01; BAQ SFit: −0.41, *p* < 0.01 T4: BAQ FF: 0.38, *p* < 0.01; BAQ Attr: −0.54, *p* < 0.01; BAQ Sal: 0.36, *p* < 0.01; BAQ SFit: −0.35, *p* < 0.01 T5: BAQ FF: 0.39, *p* < 0.01; BAQ Attr: −0.46, *p* < 0.01; BAQ Sal: 0.37, *p* < 0.01; BAQ SFit: −0.37, *p* < 0.01		5 middle
Joanne Phillips (2013) (58)	Australia	Longitudinal	126	31.00 ± 4.11	1. 3 months postpartum 2. 6 months postpartum 3. 9 months postpartum	BAQ	T1: BAQ FF: 39.60 ± 10.28 BAQ Attr: 15.92 ± 3.16 BAQ Sal: 12.78 ± 3.91 BAQ SFit: 18.00 ± 2.62 T2: BAQ FF: 36.28 ± 10.16 BAQ Attr: 15.98 ± 2.77 BAQ Sal: 12.02 ± 3.63 BAQ SFit: 19.04 ± 3.33	EPDS	T1: 6.63 ± 4.31 T2: 9.16 ± 4.65	Pearson’s r: T1: BAQ FF: 0.31, *p* < 0.01; BAQ Attr: −0.44, *p* < 0.01; BAQ Sal: 0.19, *p* < 0.05; BAQ SFit: −0.33, *p* < 0.01 T2: BAQ FF: 0.29, *p* < 0.01; BAQ Attr: −0.37, *p* < 0.01; BAQ Sal: 0.31, *p* < 0.01; BAQ SFit: −0.36, *p* < 0.01		5 middle
Rhian Collings (2018) (59)	Australia	Longitudinal	178	19–25: 9.9% 26–29: 26.9% 30–34: 39.5% 35–39: 20.5% 40–43: 2.5% (Proportion of Each Age Group)	T1: First trimester (16.97 ± 1.35 Weeks) T2: Third trimester (33.33 ± 2.05 weeks) T3: 12 months postpartum (53.12 ± 3.34 weeks)	BAQ	T2: BAQ FF: 28.17 ± 8.02 BAQ Attr: 13.03 ± 1.93 BAQ Sal: 12.61 ± 1.92 BAQ SFit: 17.49 ± 2.67 T3: BAQ FF: 33.78 ± 10.13 BAQ Attr: 15.39 ± 2.81 BAQ Sal: 13.43 ± 2.58 BAQ SFit: 16.44 ± 2.76	EPDS	T2: 18.45 ± 2.31 T3: 18.82 ± 2.06	Pearson’s r: T2: BAQ FF: −0.24, *p* < 0.05; BAQ Sal: −0.27, *p* < 0.05; T3: BAQ FF: 0.23, *p* < 0.05; BAQ Attr: −0.32, *p* < 0.05; BAQ Sal: 0.20, *p* < 0.01; BAQ SFit: −0.30, *p* < 0.05		5 middle
Sofia Rallis (2007) (63)	Australia	Longitudinal	79	32.45 ± 3.76	1. First 3 months of pregnancy 2. 32–39 weeks of pregnancy 3. 6 weeks postpartum 4. 6 months postpartum 5. 12 months postpartum	BAQ	T1: BAQ FF: 33.84 9.30 BAQ Attr: 18.01 2.55 BAQ Sal: 12.29 3.90 BAQ SFit: 21.42 ± 4.26	BDI	T4: 3.16 ± 3.14	Pearson’s *r* (Body Image in the First 3 Months of Pregnancy and Depression at 6 Months Postpartum): BAQ FF: 0.24, *p* < 0.01; BAQ Attr: 0.29, *p* < 0.05; BAQ Sal: 0.13, *p* > 0.05; BAQ SFit: −0.34, *p* < 0.05	BAQ FF: 0.25, *p* < 0.01; BAQ Attr: 0.30, *p* < 0.05; BAQ Sal: 0.14, *p* < 0.05; BAQ SFit: −0.35, *p* < 0.05	4 middle
Eliza Hartley (2018) (64)	Australia	Longitudinal	364	31.0 ± 4.6	1.6–10 weeks of pregnancy 2. 3 months postpartum 3. 6 months postpartum 4. 12 months postpartum	BAQ	/	EPDS	T2: 6.0 ± 4.3 T3: 5.9 ± 4.6 T4: 5.0 ± 4.1	Pearson’s *r* (3 Months Postpartum): BAQ FF: *r* = 0.18, *p*<0.01; BAQ Attr: *r* = −0.4, *p*<0.01; BAQ Sal: *r* = 0.16, *p*<0.05; BAQ SFit: *r* = −0.31, *p*<0.01		7 middle
Rachel F. Rodgers (2018) (60)	USA	Cross-sectional	151	32.77 ± 4.47	6 months postpartum	EDI	29.87 ± 6.88	DASS	32.75 ± 8.87	Pearson’s *r*: *r* = 0.27, *p*<0.01		5 middle

### 3.3 Quality assessment results

The quality of the 28 included studies was assessed using the 11-item AHRQ checklist. Of the 28 studies, 2 studies were rated as low quality (17, 66), and the remaining 26 were rated as moderate quality (39, 48–64, 67–73). No high-quality studies were identified. None of the studies described measures taken to ensure data quality and/or control for confounding factors, resulting in a score of 0 for these items. Among the 11 longitudinal studies, all except two (52, 58) described the completeness of follow-up data. Three studies did not report participant response rates or data collection completeness (49, 53, 70). Detailed quality assessment scores for each study are presented in Table 1 and Supplementary Table 1.

### 3.4 Meta-analysis results

**Figure 2:**
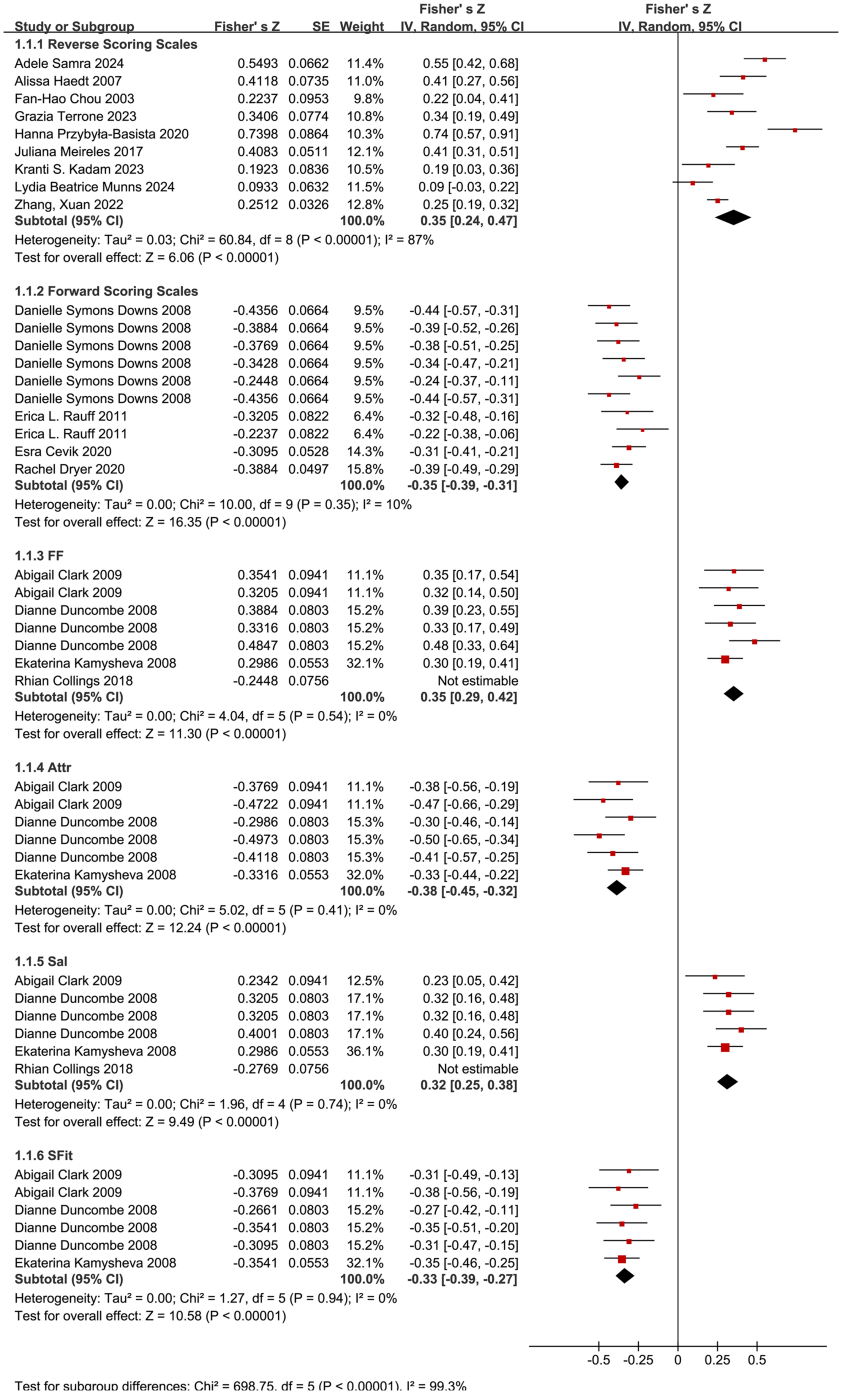
Forest plots for the pregnancy period subgroup.

**Figure 3:**
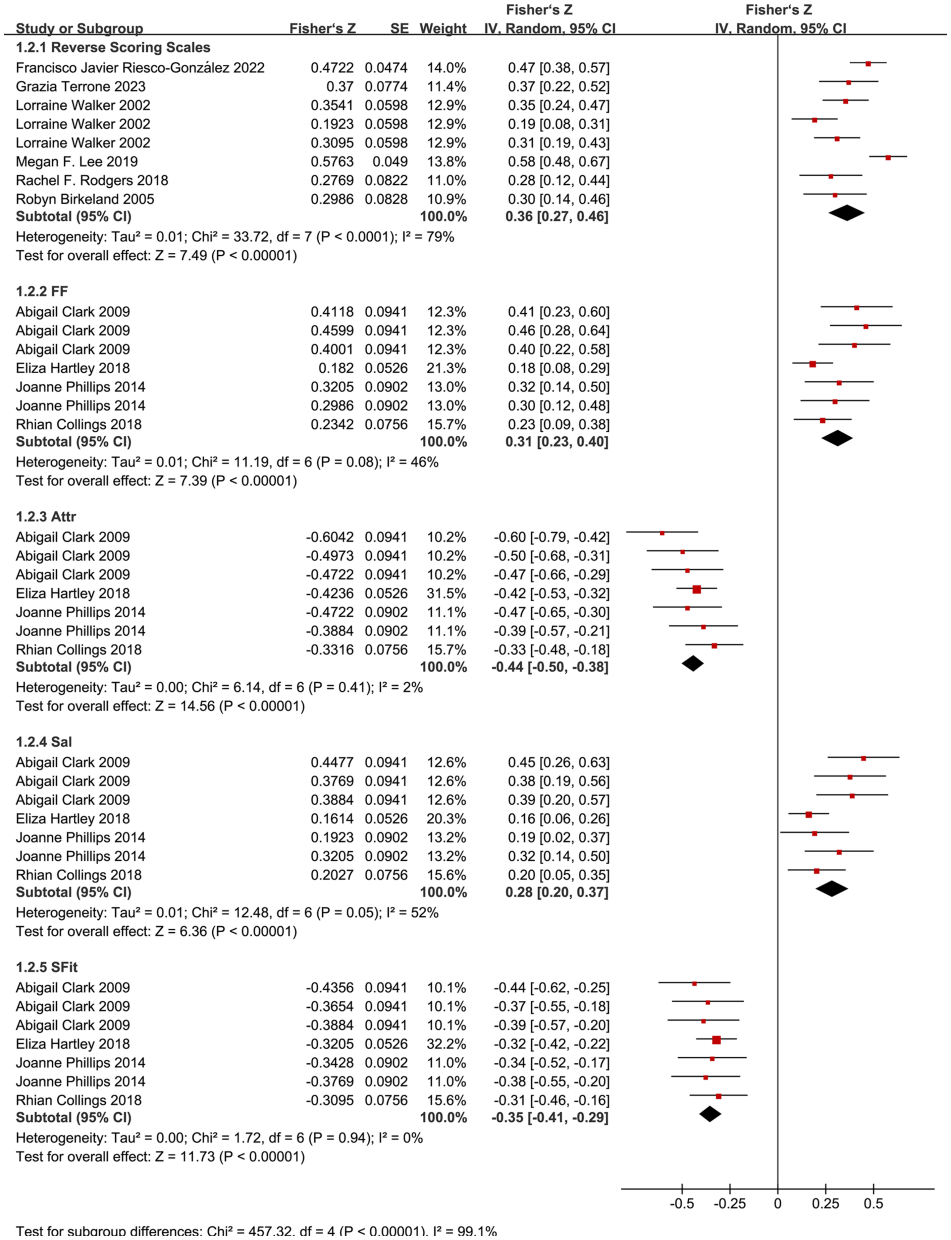
Forest plots for the postpartum period subgroup.

Subgroup analyses were conducted based on the timing of outcome measurement and the scoring direction of body image measurement tools. For studies using the BAQ, subgroup analyses were performed for its four dimensions (Feeling Fat, FF; Strength and Fitness, SFit; Salience of Weight and Shape, Sal; Attractiveness, Attr). The overall forest plot for all meta-analyses is shown in Figures 2, 3.

#### 3.4.1 Correlation between body image and depression symptoms during pregnancy

Reverse-Scoring Body Image Scales (BIPS, BCS, BSQ, EDI, BUMPs, BICI): A meta-analysis of 9 studies (48, 51, 54, 62, 65, 67, 70–72) using a random-effects model showed the following results: summary Fisher’s *Z* = 0.35 (95% CI: 0.24, 0.47; *p* < 0.01), *I*^2^ = 87% (high heterogeneity), summary *r* = 0.34 (95% CI: 0.24, 0.44). The effect size was statistically significant, indicating a moderate positive correlation between body dissatisfaction and depression during pregnancy.

Forward-Scoring Body Image Scales (BPSS, BIS, BASS): A meta-analysis of 4 studies (17, 39, 56, 68) using a random-effects model showed the following results: summary Fisher’s *Z* = −0.35 (95% CI: −0.39, −0.31; *p* < 0.01), *I*^2^ = 10% (low heterogeneity), summary *r* = −0.34 (95% CI: −0.37, −0.30). The effect size was statistically significant, indicating a moderate negative correlation between body satisfaction and depression during pregnancy.

BAQ Subgroup Analysis: After excluding outliers (details in Section 3.5 Sensitivity Analysis), the meta-results of 3 studies (55, 57, 66) were as follows:

FF: summary Fisher’s *Z* = 0.35 (95% CI: 0.29, 0.42; *p* < 0.01), *I*^2^ = 0%, summary *r* = 0.34 (95% CI: 0.28, 0.40);

Attr: summary Fisher’s *Z* = −0.38 (95% CI: −0.45, −0.32; *p* < 0.01), *I*^2^ = 0%, summary *r* = −0.36 (95% CI: −0.42, −0.31);

Sal: summary Fisher’s *Z* = 0.32 (95% CI: 0.25, 0.38; *p* < 0.01), *I*^2^ = 0%, summary *r* = 0.31 (95% CI: 0.25, 0.36);

SFit: summary Fisher’s *Z* = −0.33 (95% CI: −0.39, −0.27; *p* < 0.01), *I*^2^ = 0%, summary *r* = −0.32 (95% CI: −0.37, −0.26).

All dimensions showed statistically significant effect sizes with low heterogeneity. Perceptions of fatness and weight/shape were moderately positively correlated with depression, while perceptions of strength, fitness, and attractiveness were moderately negatively correlated with depression.

#### 3.4.2 Correlation between body image and depression symptoms during postpartum

Only one article (49) used a forward-scoring scale; a descriptive analysis was performed directly: this study reported a negative correlation between body satisfaction and postpartum depressive symptoms.

Reverse-Scoring Body Image Scales (BIPS, BCS, BSQ, EDI, BUMPs, BICI): A meta-analysis of 6 studies (50, 52, 53, 60, 61, 70) using a random-effects model showed the following results: summary Fisher’s *Z* = 0.36 (95% CI: 0.27, 0.46; *p* < 0.01), *I*^2^ = 79% (high heterogeneity), summary *r* = 0.35 (95% CI: 0.26, 0.43). The effect size was statistically significant, indicating a moderate positive correlation between body dissatisfaction and depression during the postpartum period.

BAQ Subgroup Analysis: The meta-results of 4 studies (57–59, 64) were as follows:

FF: summary Fisher’s *Z* = 0.31 (95% CI: 0.23, 0.40; *p* < 0.01), *I*^2^ = 46% (moderate heterogeneity), summary *r* = 0.30 (95% CI: 0.23, 0.38);

Attr: summary Fisher’s *Z* = −0.44 (95% CI: −0.50, −0.38; *p* < 0.01), *I*^2^ = 2%, summary *r* = −0.41 (95% CI: −0.46, −0.36);

Sal: summary Fisher’s *Z* = 0.28 (95% CI: 0.20, 0.37; *p* < 0.01), *I*^2^ = 52% (moderate heterogeneity), summary *r* = 0.27 (95% CI: 0.20, 0.35);

SFit: summary Fisher’s *Z* = −0.35 (95% CI: −0.41, −0.29; *p* < 0.01), *I*^2^ = 0%, summary *r* = −0.34 (95% CI: −0.39, −0.28).

All dimensions showed statistically significant effect sizes, with moderate heterogeneity for FF and Sal dimensions and low heterogeneity for Attr and SFit dimensions. Perceptions of fatness and weight/shape were moderately positively correlated with depression, while perceptions of strength, fitness, and attractiveness were moderately negatively correlated with depression.

### 3.5 Sensitivity analysis

Sensitivity analysis using the leave-one-out method showed that the overall meta-results were robust, and it was not performed for the “pre-pregnancy body image and depression symptoms in pregnancy” subgroup due to the small number of included studies (*n* = 2). The rest of the detailed results are presented in Supplementary Tables 2.1, 2.2.

For the FF and Sal dimensions of the BAQ during pregnancy, excluding the study by Rhian Collings (59), significantly reduced heterogeneity (FF: I^2^ from 90 to 0%; Sal: *I*^2^ from 91 to 0%) and altered the effect sizes (FF: from 0.27 [0.10, 0.45] to 0.30 [0.29, 0.42]; Sal: from 0.22 [0.02, 0.41] to 0.32 [0.25, 0.38]). Given the negative effect size in this study, contrary to the rest of the studies, combined with the sensitivity analysis results, the data from this study was classified as an outlier and excluded from the final analysis.

### 3.6 Publication bias assessment

**Figure 4:**
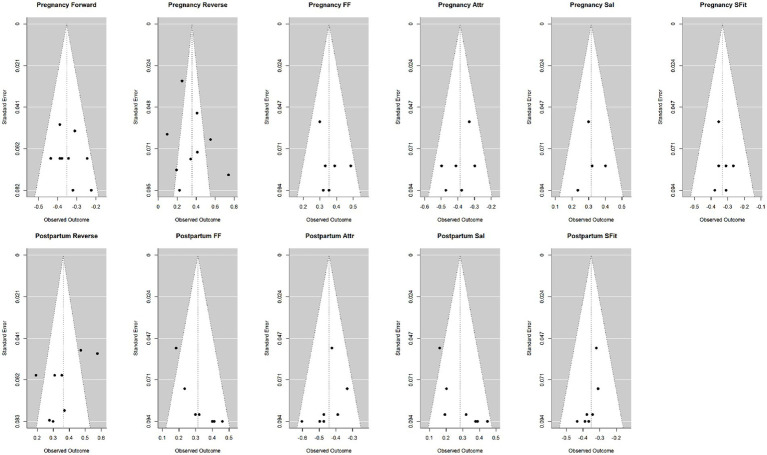
The funnel plots of each subgroup.

Funnel plots and the trim-and-fill method were used to assess publication bias. Publication bias analysis was not performed for the “pre-pregnancy body image and depression symptoms during pregnancy” subgroup (*n* = 2, insufficient for reliable funnel plot interpretation). No significant publication bias was observed for the pregnancy subgroups (0–2 missing studies). There was a slight publication bias in the postpartum subgroups (particularly for the FF dimension), which may limit the interpretation of results. The funnel plots and the results of the trim-and-fill method are provided in Figure 4 and Supplementary Table 3.

## 4 Discussion

This meta-analysis synthesized 28 studies to quantify the association between body image and perinatal depression, addressing a critical gap in prior quantitative synthesis. The results indicate a significant, moderate association between body image and perinatal depression, which is consistent with multiple previous studies and narrative reviews (17, 23–25, 27, 29, 74). Women who are more satisfied with their body image are less likely to have depression during pregnancy and postpartum, reinforcing body image disturbance as an important risk factor for perinatal depression. This study extends prior work by explicitly testing for stage-specific differences. Although previous studies suggest that postpartum women experience less protective body image compared to pregnancy and are more susceptible to pressures of achieving an ideal postpartum body shape (13), our analysis did not find a significant difference between stages (Table 2). To ensure that our findings reflect the most up-to-date evidence, we conducted a supplementary search in September 2025 for studies published after our original search period (up to December 2024). This additional search did not identify any eligible studies.

**Table 2:** Summary of meta-analysis subgroup results.

Period	Subgroup	No. of studies	Effect size (r) (95% CI)	*p*	I^2^
Pregnancy	Reverse Scoring Scales	9	0.34 (0.24, 0.44)	< 0.01	87%
	Forward Scoring Scales	4	−0.34 (−0.37, −0.30)	< 0.01	10%
	BAQ (FF)	3	0.34 (0.28, 0.40)	< 0.01	0%
	BAQ (Attr)	3	−0.36 (−0.42, −0.31)	< 0.01	0%
	BAQ (Sal)	3	0.31 (0.25, 0.36)	< 0.01	0%
	BAQ (SFit)	3	−0.32 (−0.37, −0.26)	< 0.01	0%
Postpartum	Reverse Scoring Scales	6	0.35 (0.26, 0.43)	< 0.01	79%
	BAQ (FF)	4	0.30 (0.23, 0.38)	< 0.01	46%
	BAQ (Attr)	4	−0.41 (−0.46, −0.36)	< 0.01	2%
	BAQ (Sal)	4	0.27 (0.20, 0.35)	< 0.01	52%
	BAQ (SFit)	4	−0.34 (−0.39, −0.28)	< 0.01	0%

Subgroup analyses were conducted using a random-effects model.

Subgroup analyses provided additional insights. Reverse-scored tools (pregnancy and postpartum) showed a significant positive correlation between body dissatisfaction and depression symptoms. Forward-scored tools (during pregnancy) indicated a significant negative correlation between body image satisfaction and depression symptoms. BAQ subgroup analysis further revealed dimension-specific differences in body image: perception of body fat (FF), salience of weight and body shape (Sal) showed positive correlations with depression, while perception of body attractiveness (Attr) and physical strength and fitness (SFit) correlated negatively. The subgroup analyzing pre-pregnancy body image and depression symptoms during pregnancy showed the same results, but with only 2 included studies (no sensitivity/publication bias analyses).

Body image disturbance may contribute to depression via various psychological and social pathways. Psychologically, body dissatisfaction may directly reduce self-esteem and self-worth, creating a vicious cycle where low self-esteem exacerbates negative feelings about appearance and attractiveness (12, 75). Additionally, depressive symptoms such as guilt, worthlessness, and hopelessness, as well as rumination and catastrophic thinking, can further amplify focus on perceived bodily “flaws,” deepening this cycle (76). In terms of social factors, exposure to idealized slimness can negatively affect body image and mood, while positive portrayals of bodily changes offer protection (77–79). Social support, particularly from partners, is also critical—receiving positive feedback on their bodies from partners is an overwhelmingly positive experience for women, and those whose partners are delighted with their body shape tend to show greater body satisfaction (80, 81). Family involvement more broadly may help reduce societal pressures and promote healthier adjustment to bodily changes.

Therefore, body image could be considered as part of prenatal mental health screening, and the assessment may be an effective tool for early identification of perinatal depression (25, 27, 29). However, less than one-third of professionals assess or discuss body image in routine prenatal care (82, 83), and nearly 20% of women report weight-related stigma in healthcare settings (84). As a result, a growing number of studies are calling for increased training for healthcare professionals to provide more specialized support for pregnant women (27, 85, 86), especially since over 80% of women are willing to participate in body image-focused programs (19). Prenatal courses and psychological education that emphasize normal physical changes and highlight the body’s functionality may help pregnant women reduce excessive concerns about appearance and may therefore represent promising strategies to support maternal mental health (87). Although this study provides a theoretical basis for the association between body image and depression during the perinatal period, there are some limitations. First, high heterogeneity in pregnancy (*I*^2^ = 87%) and postpartum (*I*^2^ = 79%) reverse-scoring subgroups, probably due to differences in the measurement tools, which somewhat limits the explanatory power of the results. Second, the small number of studies in some subgroups (e.g., pre-pregnancy body image) affected the generalizability of the results.

This study is the first meta-analysis to quantify the association between body image and perinatal depression, reinforcing the view that body image disturbance is an important risk factor and providing scientific evidence for clinical practice. Future research should adopt longitudinal designs or risk-based metrics (e.g., odds ratios or risk ratios) to more directly quantify the likelihood of perinatal depression associated with body dissatisfaction, thereby offering a clearer understanding of the magnitude of this risk. In addition, future studies should design and implement interventions to improve body image, which may provide new pathways for supporting maternal mental health.

## 5 Conclusion

This meta-analysis indicate a significant, moderate association between body image and perinatal depression, highlighting the importance of body image in this period. Early identification and targeted interventions may help reduce the risk of perinatal depression and improve maternal and infant health outcomes. Future research can further explore the mechanisms and develop intervention strategies to support clinical practice.

## Data Availability

The original contributions presented in the study are included in the article/Supplementary material, further inquiries can be directed to the corresponding author/s.
