# Supplementary material for: The association between body image and depressive symptoms in pregnant and postpartum women: a meta-analysis
Source: Front Public Health. 2025 Oct 8;13:1655639. doi: 10.3389/fpubh.2025.1655639 (PMC12540066; doi:10.3389/fpubh.2025.1655639)
Supplement: Supplementary file 1 [file Data_Sheet_1.DOCX]

Supplementary Material

# Supplementary Data

# Supplementary Data 1. The search strategies (take Embase as an example).

#4. #1 AND #2 AND #3

#3. 'postnatal depression'/exp OR 'puerperal disorder'/exp OR 'depression'/exp OR (((((((((((postpartum AND depression OR postnatal) AND depression OR (depression,) AND postnatal) OR perinatal) AND depression OR puerperium) AND depression OR new) AND mother AND depression OR maternal) AND depression OR postpartum) AND mood AND disorders OR depression) AND after AND childbirth OR 'post birth') AND depression OR baby) AND blues)

#2. 'body image'/exp OR 'body dissatisfaction'/exp OR ((((((body AND satisfaction OR body) AND appreciation OR body) AND concerns OR body) AND image AND disturbance OR body) AND schema* OR body) AND representation*)

#1. 'pregnancy'/exp OR pregnan* OR gravida* OR matern* OR gestation OR prenatal OR antenatal

**Supplementary Data 2. Fisher's z-transformation and inverse transformation formula.**

1. **Fisher's Z = 0.5×ln**$\frac{\boldsymbol{1+r}}{\boldsymbol{1-r}}$
2. **Vz =** $\frac{\boldsymbol{1}}{\boldsymbol{n-3}}$
3. **S_E_ =** $\sqrt{\boldsymbol{Vz}}$
4. **Summary r =** $\frac{\boldsymbol{e}^{\boldsymbol{2Z}}\boldsymbol{-1}}{\boldsymbol{e}^{\boldsymbol{2Z}}\boldsymbol{+1}}$**（Z: summary Fisher's Z）**

**Supplementary Data 3. The code for the trim-and-fill method and drawing funnel plots.**

**The code for the trim-and-fill method**:

install.packages ("readxl")

install.packages ("metafor")

library (readxl)

library (metafor)

data <- read_excel ("C:\\Users\\.xlsx")

head (data)

meta_analysis <- rma (yi = Fisher_z, sei = SE_z, data = data, method = "REML")

summary (meta_analysis)

funnel (meta_analysis)

trimfill (meta_analysis)

**The code for drawing funnel plot:**

install.packages ("readxl")

install.packages ("metafor")

library (readxl)

library (metafor)

png ("forest_plots_high_res.png", width = 5000, height = 3000, res = 300)

par (mfrow = c (2, 6), oma = c (2, 2, 2, 2), mar = c (4, 4, 4, 4))

data_forward <- read_excel ("C:\\Users\\.xlsx")

meta_forward <- rma (yi = Fisher_z, sei = SE, data = data_forward, method = "REML")

funnel (meta_forward, main = " Pregnancy Forward ")

data_reverse <- read_excel ("C:\\Users\\.xlsx")

meta_reverse <- rma (yi = Fisher_z, sei = SE, data = data_reverse, method = "REML")

funnel (meta_reverse, main = " Pregnancy Reverse ")

data_FF <- read_excel ("C:\\Users\\.xlsx")

meta_FF <- rma (yi = Fisher_z, sei = SE, data = data_FF, method = "REML")

funnel (meta_FF, main = " Pregnancy FF")

data_Attr <- read_excel ("C:\\Users\\.xlsx")

meta_Attr <- rma (yi = Fisher_z, sei = SE, data = data_Attr, method = "REML")

funnel (meta_Attr, main = " Pregnancy Attr")

data_Sal <- read_excel ("C:\\Users\\.xlsx")

meta_Sal <- rma (yi = Fisher_z, sei = SE, data = data_Sal, method = "REML")

funnel (meta_Sal, main = " Pregnancy Sal")

data_SFit <- read_excel ("C:\\Users\\.xlsx")

meta_SFit <- rma (yi = Fisher_z, sei = SE, data = data_SFit, method = "REML")

funnel (meta_SFit, main = " Pregnancy SFit")

data_post_reverse <- read_excel ("C:\\Users\\.xlsx")

meta_post_reverse <- rma (yi = Fisher_z, sei = SE, data = data_post_reverse, method = "REML")

funnel (meta_post_reverse, main = " Postpartum Reverse ")

data_post_FF <- read_excel ("C:\\Users\\.xlsx")

meta_post_FF <- rma (yi = Fisher_z, sei = SE, data = data_post_FF, method = "REML")

funnel (meta_post_FF, main = " Postpartum FF")

data_post_Attr <- read_excel ("C:\\Users\\.xlsx")

meta_post_Attr <- rma (yi = Fisher_z, sei = SE, data = data_post_Attr, method = "REML")

funnel (meta_post_Attr, main = " Postpartum Attr")

data_post_Sal <- read_excel ("C:\\Users\\.xlsx")

meta_post_Sal <- rma (yi = Fisher_z, sei = SE, data = data_post_Sal, method = "REML")

funnel (meta_post_Sal, main = " Postpartum Sal")

data_post_SFit <- read_excel ("C:\\Users\\.xlsx")

meta_post_SFit <- rma (yi = Fisher_z, sei = SE, data = data_post_SFit, method = "REML")

funnel (meta_post_SFit, main = " Postpartum SFit")

dev.off ()

# Supplementary Tables

# Supplementary Table 1. The quality assessment results of included studies.

**Supplementary Table 1 provides detailed information on the quality assessment results of included studies. Due to its large size, it has been uploaded as another supplementary file (see Supplementary Table 1).**

**Supplementary Tables 2.1. The results of the sensitivity analysis for pregnancy group (Leave-One-Out Method).**

| Excluded Studies | Effect Size After Exclusion (Fisher z) | I² After Exclusion | p-value After Exclusion |
| --- | --- | --- | --- |
| Reverse Scoring Scales | | | |
| Overall Pooled Results | 0.35 [0.24, 0.47] | 87% | p<0.01 |
| Adele Samra 2024 | 0.33 [0.21, 0.45] | 85% | p<0.01 |
| Alissa Haedt 2007 | 0.35 [0.22, 0.47] | 88% | p<0.01 |
| Fan-Hao Chou 2003 | 0.37 [0.25, 0.49] | 88% | p<0.01 |
| Grazia Terrone 2023 | 0.36 [0.23, 0.48] | 88% | p<0.01 |
| Hanna Przybyła-Basista 2020 | 0.31 [0.21, 0.41] | 81% | p<0.01 |
| Juliana Meireles 2017 | 0.35 [0.22, 0.48] | 88% | p<0.01 |
| Kranti S. Kadam 2023 | 0.37 [0.25, 0.50] | 88% | p<0.01 |
| Lydia Beatrice Munns 2024 | 0.39 [0.27, 0.50] | 85% | p<0.01 |
| Zhang, Xuan 2022 | 0.37 [0.23, 0.51] | 87% | p<0.01 |
| Forward Scoring Scales | | | |
| Overall Pooled Results | -0.35 [-0.39, -0.31] | 10% | p<0.01 |
| Danielle Symons Downs 2008 | -0.36 [-0.41, -0.32] | 0 | p<0.01 |
| Danielle Symons Downs 2008 | -0.34 [-0.39, -0.30] | 3% | p<0.01 |
| Danielle Symons Downs 2008 | -0.34 [-0.39, -0.30] | 3% | p<0.01 |
| Danielle Symons Downs 2008 | -0.35 [-0.39, -0.30] | 17% | p<0.01 |
| Danielle Symons Downs 2008 | -0.35 [-0.40, -0.30] | 19% | p<0.01 |
| Danielle Symons Downs 2008 | -0.35 [-0.40, -0.31] | 20% | p<0.01 |
| Erica L. Rauff 2011 | -0.35 [-0.40, -0.31] | 19% | p<0.01 |
| Erica L. Rauff 2011 | -0.36 [-0.40, -0.32] | 0 | p<0.01 |
| Esra Cevik 2020 | -0.36 [-0.41, -0.31] | 13% | p<0.01 |
| Rachel Dryer 2020 | -0.35 [-0.39, -0.30] | 15% | p<0.01 |
| BAQ (FF) | | | |
| Overall Pooled Results | 0.27 [0.10, 0.45] | 90% | p=0.003 |
| Rhian Collings 2018 | 0.35 [0.29, 0.42] | 0% | p<0.01 |
| Ekaterina Kamysheva 2008 | 0.27 [0.05, 0.50] | 91% | p=0.02 |
| Dianne Duncombe 2008 | 0.26 [0.05, 0.46] | 91% | p=0.01 |
| Dianne Duncombe 2008 | 0.27 [0.06, 0.48] | 91% | p=0.01 |
| Dianne Duncombe 2008 | 0.24 [0.04, 0.43] | 90% | p=0.02 |
| Abigail Clark 2009 | 0.26 [0.06, 0.47] | 91% | p=0.01 |
| Abigail Clark 2009 | 0.27 [0.06, 0.47] | 91% | p=0.01 |
| BAQ (Attr) | | | |
| Overall Pooled Results | -0.38 [-0.45, -0.32] | 0% | p<0.01 |
| Abigail Clark 2009 | -0.39 [-0.46, -0.32] | 20% | p<0.01 |
| Abigail Clark 2009 | -0.37 [-0.44, -0.31] | 1% | p<0.01 |
| Dianne Duncombe 2008 | -0.36 [-0.43, -0.30] | 0 | p<0.01 |
| Dianne Duncombe 2008 | -0.38 [-0.46, -0.31] | 18% | p<0.01 |
| Dianne Duncombe 2008 | -0.40 [-0.47, -0.33] | 0 | p<0.01 |
| Ekaterina Kamysheva 2008 | -0.41 [-0.48, -0.34] | 0 | p<0.01 |
| BAQ (Sal) | | | |
| Overall Pooled Results | 0.22 [0.02, 0.41] | 91% | p=0.03 |
| Abigail Clark 2009 | 0.21 [-0.02, 0.45] | 93% | p=0.08 |
| Dianne Duncombe 2008 | 0.20 [-0.04, 0.43] | 92% | p=0.11 |
| Dianne Duncombe 2008 | 0.20 [-0.04, 0.43] | 92% | p=0.11 |
| Dianne Duncombe 2008 | 0.18 [-0.05, 0.41] | 92% | p=0.12 |
| Ekaterina Kamysheva 2008 | 0.20 [-0.06, 0.45] | 92% | p=0.13 |
| Rhian Collings 2018 | 0.32 [0.25, 0.38] | 0 | p<0.01 |
| BAQ (SFit ) | | | |
| Overall Pooled Results | -0.33 [-0.39, -0.27] | 0 | p<0.01 |
| Abigail Clark 2009 | -0.33 [-0.39, -0.26] | 0 | p<0.01 |
| Abigail Clark 2009 | -0.33 [-0.40, -0.27] | 0 | p<0.01 |
| Dianne Duncombe 2008 | -0.34 [-0.41, -0.28] | 0 | p<0.01 |
| Dianne Duncombe 2008 | -0.33 [-0.39, -0.26] | 0 | p<0.01 |
| Dianne Duncombe 2008 | -0.34 [-0.40, -0.27] | 0 | p<0.01 |
| Ekaterina Kamysheva 2008 | -0.32 [-0.40, -0.25] | 0 | p<0.01 |

**Supplementary Tables 2.2. The results of the sensitivity analysis for postpartum group (Leave-One-Out Method).**

| Excluded Studies | Effect Size After Exclusion (Fisher z) | I² After Exclusion | p-value After Exclusion |
| --- | --- | --- | --- |
| Reverse Scoring Scales | | | |
| Overall Pooled Results | 0.36 [0.27, 0.46] | 79% | p<0.01 |
| Francisco Javier Riesco-González 2022 | 0.34 [0.24, 0.45] | 80% | p<0.01 |
| Grazia Terrone 2023 | 0.36 [0.25, 0.47] | 82% | p<0.01 |
| Lorraine Walker 2002 | 0.36 [0.25, 0.47] | 82% | p<0.01 |
| Lorraine Walker 2002 | 0.39 [0.30, 0.48] | 72% | p<0.01 |
| Lorraine Walker 2002 | 0.37 [0.26, 0.48] | 81% | p<0.01 |
| Megan F. Lee 2019 | 0.33 [0.25, 0.41] | 60% | p<0.01 |
| Rachel F. Rodgers 2018 | 0.37 [0.27, 0.47] | 81% | p<0.01 |
| Robyn Birkeland 2005 | 0.37 [0.27, 0.47] | 82% | p<0.01 |
| BAQ (FF) | | | |
| Overall Pooled Results | 0.31 [0.23, 0.40] | 46% | p<0.01 |
| Abigail Clark 2009 | 0.30 [0.21, 0.39] | 48% | p<0.01 |
| Abigail Clark 2009 | 0.30 [0.21, 0.39] | 47% | p<0.01 |
| Abigail Clark 2009 | 0.29 [0.21, 0.37] | 34% | p<0.01 |
| Eliza Hartley 2018 | 0.34 [0.27, 0.41] | 0 | p<0.01 |
| Joanne Phillips 2014 | 0.32 [0.22, 0.41] | 55% | p<0.01 |
| Joanne Phillips 2014 | 0.32 [0.22, 0.42] | 55% | p<0.01 |
| Rhian Collings 2018 | 0.33 [0.23, 0.43] | 52% | p<0.01 |
| BAQ (Attr) | | | |
| Overall Pooled Results | -0.44 [-0.50, -0.38] | 2% | p<0.01 |
| Abigail Clark 2009 | -0.44 [-0.50, -0.37] | 13% | p<0.01 |
| Abigail Clark 2009 | -0.44 [-0.51, -0.37] | 17% | p<0.01 |
| Abigail Clark 2009 | -0.42 [-0.48, -0.36] | 0 | p<0.01 |
| Eliza Hartley 2018 | -0.45 [-0.53, -0.37] | 16% | p<0.01 |
| Joanne Phillips 2014 | -0.44 [-0.51, -0.37] | 17% | p<0.01 |
| Joanne Phillips 2014 | -0.45 [-0.52, -0.38] | 13% | p<0.01 |
| Rhian Collings 2018 | -0.46 [-0.53, -0.40] | 0 | p<0.01 |
| BAQ (Sal) | | | |
| Overall Pooled Results | 0.28 [0.20, 0.37] | 52% | p<0.01 |
| Abigail Clark 2009 | 0.26 [0.17, 0.34] | 39% | p<0.01 |
| Abigail Clark 2009 | 0.27 [0.18, 0.37] | 54% | p<0.01 |
| Abigail Clark 2009 | 0.27 [0.18, 0.36] | 52% | p<0.01 |
| Eliza Hartley 2018 | 0.31 [0.23, 0.40] | 29% | p<0.01 |
| Joanne Phillips 2014 | 0.30 [0.20, 0.40] | 58% | p<0.01 |
| Joanne Phillips 2014 | 0.28 [0.18, 0.38] | 58% | p<0.01 |
| Rhian Collings 2018 | 0.30 [0.20, 0.40] | 57% | p<0.01 |
| BAQ (SFit ) | | | |
| Overall Pooled Results | -0.35 [-0.41, -0.29] | 0 | p<0.01 |
| Abigail Clark 2009 | -0.34 [-0.40, -0.28] | 0 | p<0.01 |
| Abigail Clark 2009 | -0.35 [-0.41, -0.29] | 0 | p<0.01 |
| Abigail Clark 2009 | -0.35 [-0.41, -0.28] | 0 | p<0.01 |
| Eliza Hartley 2018 | -0.36 [-0.44, -0.29] | 0 | p<0.01 |
| Joanne Phillips 2014 | -0.35 [-0.41, -0.29] | 0 | p<0.01 |
| Joanne Phillips 2014 | -0.35 [-0.41, -0.29] | 0 | p<0.01 |
| Rhian Collings 2018 | -0.36 [-0.42, -0.29] | 0 | p<0.01 |

**Supplementary Tables 3. The results of publication bias analysis (Trim-and-Fill Method Results).**

| Subgroup | Number of Missing Studies | Adjusted Effect Size | 95% CI | *p* | I^2^ (%) | τ^2^ |
| --- | --- | --- | --- | --- | --- | --- |
| Body Image and Depression During Pregnancy | | | | | | |
| Reverse Scoring | 0 | 0.36 | (0.23，0.48 ) | <.0001 | 89.28% | 0.0325 |
| Forward Scoring | 0 | -0.35 | (-0.39，-0.31 ) | <.0001 | 4.29% | 0.0002 |
| FF | 0 | 0.35 | (0.29，0.42) | <.0001 | 0 | 0 |
| Attr | 2 | -0.35 | (-0.42，-0.28) | <.0001 | 39.08% | 0.0041 |
| Sal | 0 | 0.32 | (0.25，0.38) | <.0001 | 0 | 0 |
| SFit | 0 | -0.33 | (-0.39，-0.27) | <.0001 | 0 | 0 |
| Body Image and Depression During Postpartum | | | | | | |
| Reverse Scoring | 2 | 0.4 | (0.31，0.49 ) | <.0001 | 80.93% | 0.0167 |
| FF | 4 | 0.22 | (0.13，0.32 ) | <.0001 | 72.76% | 0.019 |
| Attr | 2 | -0.41 | (-0.48，-0.35 ) | <.0001 | 28.12% | 0.0027 |
| Sal | 2 | 0.24 | (0.15，0.33 ) | <.0001 | 64.77% | 0.0127 |
| SFit | 3 | -0.33 | (-0.38，-0.28 ) | <.0001 | 0 | 0 |
