# Supplementary material for: The association between body image and depressive symptoms in pregnant and postpartum women: a meta-analysis
Source: Front Public Health. 2025 Oct 8;13:1655639. doi: 10.3389/fpubh.2025.1655639 (PMC12540066; doi:10.3389/fpubh.2025.1655639)
Supplement: Supplementary file 2 [file Table_1.DOCX]

Supplementary Table 1. The quality assessment results of included studies (n = 28).

| Study | 1. Define the source of information (survey, record review). | 2. List inclusion and exclusion criteria for exposed and unexposed subjects (cases and controls) or refer to previous publications. | 3. Indicate time period used for identifying patients. | 4. Indicate whether or not subjects were consecutive if not population-based. | 5. Indicate if evaluators of subjective components of study were masked to other aspects of the participants. | 6. Describe any assessments undertaken for quality assurance purposes (e.g., test/retest of primary outcome measurements). | 7. Explain any patient exclusions from analysis. | 8. Describe how confounding was assessed and/or controlled. | 9. If applicable, explain how missing data were handled in the analysis. | 10. Summarize patient response rates and completeness of data collection. | 11. Clarify what follow-up, if any, was expected and the percentage of patients for which incomplete data or follow-up was obtained. | Total Score | Grade |
| --- | --- | --- | --- | --- | --- | --- | --- | --- | --- | --- | --- | --- | --- |
| Adele Samra 2024 | yes | yes | no | yes | Unclear | no | yes | no | yes | yes | Not applicable | 6 | middle |
| Mei-Ling Chen 2023 | yes | yes | yes | yes | Unclear | no | yes | no | Unclear | Unclear | Not applicable | 5 | middle |
| Francisco Javier Riesco-González 2022 | yes | yes | yes | no | Unclear | no | no | no | Unclear | yes | Not applicable | 4 | middle |
| Fan-Hao Chou 2003 | yes | yes | no | yes | Unclear | no | yes | no | Unclear | yes | Not applicable | 5 | middle |
| Robyn Birkeland 2005 | yes | yes | no | yes | Unclear | no | yes | no | Unclear | Unclear | Not applicable | 4 | middle |
| Alissa Haedt 2007 | yes | no | no | yes | Unclear | no | yes | no | yes | yes | Not applicable | 5 | middle |
| Ekaterina Kamysheva 2008 | yes | yes | no | yes | Unclear | no | yes | no | Unclear | yes | Not applicable | 5 | middle |
| Rachel F. Rodgers 2018 | yes | yes | yes | yes | Unclear | no | no | no | Unclear | yes | Not applicable | 5 | middle |
| Rachel Dryer 2020 | yes | no | no | yes | Unclear | no | no | no | Unclear | yes | Not applicable | 3 | low |
| Megan F. Lee 2019 | yes | yes | no | yes | Unclear | no | yes | no | yes | yes | Not applicable | 6 | middle |
| Hanna Przybyła-Basista 2020 | yes | yes | yes | no | Unclear | no | yes | no | yes | yes | Not applicable | 6 | middle |
| Kranti S. Kadam 2023 | yes | yes | no | no | Unclear | no | yes | no | yes | yes | Not applicable | 5 | middle |
| Esra Cevik 2020 | yes | yes | yes | no | Unclear | no | yes | no | Unclear | yes | Not applicable | 5 | middle |
| Lydia Beatrice Munns  2024 | yes | yes | yes | no | Unclear | no | yes | no | Unclear | yes | Not applicable | 5 | middle |
| Grazia Terrone 2023 | yes | yes | yes | no | Unclear | no | yes | no | Unclear | no | Not applicable | 4 | middle |
| Juliana Meireles 2017 | yes | yes | yes | yes | Unclear | no | yes | no | yes | yes | Not applicable | 7 | middle |
| Zhang, Xuan 2022 | yes | yes | yes | no | Unclear | no | yes | no | Unclear | yes | Not applicable | 5 | middle |
| Lorraine Walker 2002 | yes | yes | yes | yes | unclear | no | yes | no | Unclear | yes | unclear | 6 | middle |
| Danielle Symons Downs 2008 | yes | no | yes | yes | unclear | no | yes | no | Unclear | yes | yes | 6 | middle |
| Abigail Clark 2009 | yes | yes | no | no | unclear | no | yes | no | Unclear | yes | yes | 5 | middle |
| Joanne Phillips 2014 | yes | no | no | yes | unclear | no | yes | no | yes | yes | unclear | 5 | middle |
| Rhian Collings 2018 | yes | no | no | no | unclear | no | yes | no | yes | yes | yes | 5 | middle |
| Sofia Rallis 2007 | yes | no | no | no | unclear | no | yes | no | Unclear | yes | yes | 4 | middle |
| Eliza Hartley 2018 | yes | yes | yes | no | unclear | no | yes | no | yes | yes | yes | 7 | middle |
| Dianne Duncombe 2008 | yes | no | no | no | unclear | no | no | no | Unclear | yes | yes | 3 | low |
| Erica L. Rauff 2011 | yes | no | no | no | unclear | no | yes | no | yes | yes | yes | 5 | middle |
| Helen Skouteris 2005 | yes | yes | no | no | unclear | no | yes | no | yes | yes | yes | 6 | middle |
| Yang, Yiyun 2024 | yes | yes | yes | no | unclear | no | yes | no | Unclear | yes | yes | 6 | middle |
